# Trajectories and influencing factors in adolescent procrastination behavior throughout the COVID-19 pandemic: a four-wave prospective longitudinal study

**DOI:** 10.3389/fpsyg.2023.1168463

**Published:** 2023-06-22

**Authors:** Yongmei Wu, Tianyi Bu, Yunjia Xie, Ping Wei, Jinxin Zhao, Lu Chen, Kexin Qiao, Yan Wang, Jiarun Yang, Xiaohui Qiu, Xiuxian Yang, Jiawei Zhou, Zhengxue Qiao, Yanjie Yang

**Affiliations:** ^1^Psychology and Health Management Center, Harbin Medical University, Harbin, China; ^2^Beijing Hospital, Beijing, China; ^3^Department of Psychology, School of Education of Heilongjiang University, Harbin, China

**Keywords:** COVID-19, adolescent, procrastination, latent growth curve model, latent growth mixture model, prospective study

## Abstract

**Background:**

Despite the growing attention given to adolescent behavior problems, little is known about the trajectories and factors that have influenced adolescent procrastination during the COVID-19 pandemic. This study monitors changes in procrastination behavior among Chinese adolescents during the pandemic and identifies vulnerable groups.

**Methods:**

A four-wave study using a representative sample of 11-to 18-year-olds in China was conducted, with baseline data collected in June 2020 (*n* = 4,156; 49% girls) and follow-ups in December 2020 (*n* = 3,392; 50% girls), August 2021 (*n* = 2,380; 48% girls), and October 2021 (*n* = 1,485; 49% girls). Procrastination behavior was assessed using the General Procrastination Scale. Latent growth curve models, latent growth mixture modes, and multivariate logistic regression models were used to describe the trajectory of procrastination and identify predictors of deterioration.

**Results:**

The proportion and overall trends of adolescent procrastination increased with the pandemic. Higher parental over-protection was a contributing factor to the higher baseline levels leading to the faster growth of adolescent procrastination. The model identified three distinct trajectories of low-increasing [including 2,057 participants (49.5%)], moderate-stable [including 1,879 participants (45.2%)], and high-decreasing procrastination [including 220 participants (5.3%)]. More daily leisure screen-time, lower frequency of exercise weekly, and dissatisfaction with distance learning were the top three risk factors for moderate-stable and high-decreasing procrastination compared to low-increasing procrastination. Adolescents with mothers with a higher level of education were more liable to be high-decreasing procrastination than moderate-stable procrastination.

**Conclusion:**

The proportion and overall trends of adolescent procrastination increased with the pandemic. The categories of procrastination among adolescents during that time period were probed. Also, the study further clarified the risk factors for severe and moderate procrastination relative to no procrastination. Thus, effective procrastination prevention and intervention strategies need to be implemented to support adolescents, particularly those at risk.

## Introduction

In December 2019, COVID-19 suddenly erupted in China and gradually spread around the world. The World Health Organization (WHO) declared the outbreak to be a global pandemic on March 11, 2020 (Cucinotta and Vanelli, 2020). Due to the COVID-19 pandemic, higher educational institutions worldwide switched to emergency distance learning in early 2020. Stay-at-home and quarantine orders issued by governments led to the largest enforced isolation period in human history. In order to curb the spread of the virus, a lot of countries took measures to close schools and switch to distance learning or online learning (Hafstad et al., 2022). There is no doubt that adolescents are a particularly vulnerable group with immature minds and coping skills (Steinberg and Morris, 2001; Crocetti, 2017). The structural environment of distance learning is poor, and if adolescents are not able to regulate their learning and motivation more independently, they are prone to exhibit psychological and behavioral problems, including procrastination, which has not been much noticed in the context of the pandemic.

Procrastination is the voluntary postponement of starting or completing a planned behavior, despite the foreseeable negative consequences of doing so (Lay, 1986; Diotaiuti et al., 2021a). According to most research, 40–60% of college students procrastinate to a moderate or high degree (Onwuegbuzie, 2004). Procrastination is one of the most common and serious behavioral problems in adolescents, which is caused by a variety of factors. Adolescents’ procrastination can affect their mental health and have a negative impact on adulthood (Duru and Balkis, 2017). Recent studies (Albursan et al., 2022) have shown that male students, mobile phone addiction, and physical inactivity are positively associated with academic procrastination in the context of COVID-19.

However, the vast majority of these studies so far have been cross-sectional, relying primarily on college students sampling (Aznar-Díaz et al., 2020; Pelikan et al., 2021; Shi et al., 2021). With studies on adolescent’s procrastination during the pandemic being few (Chen, 2017), continuous prospective investigation of the long-term changes in adolescent’s procrastination is necessary during several waves of subsequent infection mitigation measures.

Based on previous studies and gaps, a longitudinal follow-up study of Chinese adolescents was conducted at four time points. Our study aimed to: (1) explore the status quo and overall trajectory of adolescent procrastination during the pandemic and the influencing factors of the initial level and development rate; and (2) determine the categories of procrastination trajectories during this period and the populations which exhibit more serious procrastination.

## Methods

### Study design and participants

The study is a large prospective study on adolescent procrastination behavior during the COVID-19 pandemic in China. The survey was conducted in four waves through an online survey website.^1^ Wave 1 (June 2020) was just when the pandemic leveled off and face-to-face learning resumed. Wave 2 (December 2020) was when the pandemic control was relatively stable and relaxed. Wave 3 (August 2021) was repeated outbreaks within the summer vacation period. Wave 4 (October 2021) took place when the pandemic slightly affected the region leading to a switch to distance learning. The sample is representative of the population in the north of China, a simple random sample of adolescents of key and non-key schools, public and private schools, urban and rural schools, and 11 selected middle and high schools for a comprehensive survey and follow-up. The questionnaires were available in Chinese.

The number of participants in the four surveys were 11,226, 10,550, 5,939, and 5,107, respectively. To examine the trajectories of procrastination in relation to specific measures relating to the pandemic, we focused solely on participants who had at least one initial and any subsequent repeat measurement within the four waves. These criteria provided us with data from 5,551 respondents who were followed up to complete at least two and up to four waves since the beginning of the survey. In total, 925 (22.26%) participants completed all COVID-19 web surveys, 1,251 (30.10%) completed only three surveys, and 1,980 (47.64%) completed only two surveys, as shown in Supplementary Table S1 of the Supplemental materials. Of these participants, 1,395 (25%) withheld GPS-20 data or tended not to self-identify with demographic factors and were therefore excluded from our analysis (the demographics of these participants are shown in Supplementary Table S2 of Supplemental materials), resulting in a final analytic sample size of 4,156.

This study was approved by the Ethics Committee of Harbin Medical University (HMUIRB20200002) and all participants gave informed consent.

### Measures

#### General demographic variables

The survey include gender (female = 2; male = 1), age (11–18 years old), grades (junior one to four and senior one to three, coded as 1–7, respectively), presence of siblings (siblings = 2; no siblings = 1), household economy (“Very poor” to “Very good,” coded as 1–5), and maternal education (“Primary and below” to “Masters or above,” coded from 1–6).

#### Procrastination

We calculated a composite score from summing items in the General Procrastination Scale (GPS; Lay, 1986), a 20-item tool (e.g., “When preparing for deadlines, I often waste time doing other things”) which is validated as a unidimensional measure of procrastination in different situations with 5-point responses ranging from “strongly disagree” to “totally agree.” Scores of 0–53 are thought to represent no procrastination, 53–63 moderate procrastination, and more than 63 severe procrastination. A total score was derived for each wave (20–100). Cronbach’s α coefficient was 0.739–0.847 in the four waves of this study.

#### Passive coping

The Simplified Coping Style Questionnaire (SCSQ) measures people’s attitudes and ways of coping with various life events in their daily lives and consists of 20 questions. The questionnaire has two domains, positive coping style (entries 1–12) and negative coping style (entries 13–20). In Wave 1, adolescents responded to the negative coping style domain of SCSQ using a 4-point severity/frequency scale ranging from 0 (“Never”) to 3 (“Always”; Folkman and Lazarus, 1988). An overall score was obtained by summing the scores for each item with a potential total score between 0 and 24. Cronbach’s α coefficient was 0.855 in this study.

#### Goal focused and emotional control

The Resilience Scale for Chinese Adolescents (RSCA) consists of 27 items, divided into five domains: goal focus, emotional control, positive cognition, family support, and interpersonal assistance (Hu and Gan, 2008). In Wave 1, adolescents responded to the goal focused and emotional control domains from RSCA. Each statement was rated using a 5-point Likert scale ranging from 0 (“not at all”) to 5 (“certainly true”). In this study, Cronbach’s α coefficient of the goal focused and emotional control domains were 0.891 and 0.823, respectively.

#### Emotional warmth and over-protection

The revision of the short-form Egna Minnenav Barndoms Uppfostran (s-EMBU) consists of 21 questions and includes three domains (rejection, emotional warmth, and over-protection) with the 15th question being called the reverse rating (Perris et al., 1980; Jiang et al., 2010). In Wave 1, adolescents responded to the emotional warmth and over-protection subscales from the s-EMBU. Each statement was rated using a 4-point Likert scale ranging from 1 (“Never”) to 4 (“Always”). In this study, Cronbach’s α coefficient of the emotional warmth and over-protection subscales were 0.906 and 0.752, respectively.

#### Lifestyle behaviors under the pandemic

The survey includes satisfaction of distance education (dissatisfaction of distance learning = 2; satisfaction = 1), frequent weekly exercise (barely =1; < 3 times = 2; ≥ 3 times = 3), leisure screen-time daily (< 3 h = 1; 3–5 h = 2; >5 h = 3) and daily online communication with peers (barely = 1; 0–30 min = 2; ≥ 30 min = 3) in Wave 1.

### Statistical analysis

Analyses were performed using SPSS 22.0 and Mplus 8.3 (Muthen and Muthen, 2000). We used the unspecified latent growth curve (LGC) model to investigate the change in procrastination scores over time, which allows the shape of growth trajectories to be determined from the data by using free time scores. In the model, the intercept (representing the baseline data) and the slope (representing the change over time) of the dependent variables were modeled. Missing data were addressed using full information maximum likelihood estimation (FIML), which uses all information available from all respondents, thus being less prone to biases than a complete case analysis with listwise deletion where the loss of information is larger and would lead to greater biases in estimates. The number of random start values was 200 and the maximum number of iterations was 500. To determine a good statistical fit, we accepted models that had a Comparative Fit Index (CFI) and Tucker-Lewis Index (TLI) > 0.90 and a root mean square error of approximation (RMSEA) < 0.08 (Browne and Cudeck, 1993; Wen et al., 2004). These models analyzed whether the change in the procrastination scales was predicted by individual and family-related risk factors.

Group-based trajectories of procrastination were estimated using the latent growth mixture (LGM) model. This data-driven modeling technique enables the identification of individuals with similar trajectories of procrastination by computing classes (or trajectories) of mean values within homogenous subgroups over time. We ran models with an increasing number of trajectories until non-convergence was reached. Model fit was evaluated using Bayesian information criterion (BIC), Adjusted BIC (aBIC), the Akaike information criteria (AIC), entropy index, and the Lo, Mendell, and Rubin statistic (Yungtai et al., 2001). Next, multivariate logistic regression models were fitted to examine the associations of sociodemographic, individual, and family-related risk factors with group-based procrastination trajectories. The LGM models were fitted using robust maximum likelihood estimation to account for missing data and for the non-normal distribution of the procrastination scores. The results of all multivariate logistic regression analyses are presented as adjusted odds ratios (ORs) with corresponding 95% CIs. *p* values were two-sided, and statistical significance was set at *p* < 0.05.

## Results

### Sociodemographic characteristics and the proportion of procrastination

Table 1 provides an overview of the sample characteristics (Wave 1 – Wave 4). Our analytic sample comprised 4,156 participants aged 11–18 years old (mean = 13.55; standard deviation = 1.18; 49.25% female). The percentage of adolescents reporting their procrastination increased significantly from 39.50 to 46.94% to 51.85 to 47.47% in Waves 1 – 4, respectively, but there was no significant difference between Wave 3 and Wave 4.

**Table 1:** Demographic and baseline information for participants included in the analyses.

	Wave1	Wave2	Wave3	Wave4
	4,156	3,392	2,380	1,485
Gender				
Male	2,109 (50.75)	1,701 (50.15)	1,239 (52.06)	757 (50.98)
Female	2,047 (49.25)	1,691 (49.85)	1,141 (47.94)	728 (49.02)
Age, year				
11–12	738 (17.75)	467 (13.76)	560 (23.53)	350 (23.56)
13–14	2,671 (64.27)	2,245 (66.19)	1,665 (69.96)	1,103 (74.28)
15–16	654 (15.74)	606 (17.87)	137 (5.76)	31 (2.09)
17–18	93 (2.24)	74 (2.18)	18 (0.75)	1 (0.07)
Grades				
Junior one	1,450 (34.88)	925 (27.28)	1,055 (44.33)	533 (35.89)
Junior two	1,579 (38.00)	1,372 (40.46)	1,277 (53.66)	933 (62.83)
Junior three	655 (15.76)	652 (19.23)	21 (0.88)	14 (0.94)
Junior four	191 (4.60)	188 (5.55)	1 (0.04)	4 (0.27)
Senior one	269 (6.47)	244 (7.13)	26 (1.09)	–
Senior two	10 (0.24)	9 (0.28)	–	1 (0.07)
Senior three	2 (0.05)	2 (0.07)	–	–
Presence of siblings				
No siblings	3,002 (72.23)	2,488 (73.35)	1,750 (73.53)	1,110 (74.75)
Siblings	1,154 (27.77)	904 (26.65)	630 (26.47)	375 (25.25)
Household economy				
Very poor	40 (0.96)	30 (0.88)	26 (1.08)	9 (0.60)
Poor	144 (3.45)	116 (3.42)	82 (3.45)	44 (2.96)
General	2,820 (67.85)	2,299 (67.78)	1,614 (67.82)	983 (67.20)
Good	933 (22.44)	780 (23.00)	525 (22.06)	362 (23.38)
Very good	219 (5.30)	167 (4.92)	133 (5.59)	87 (5.86)
Maternal education				
Primary and below	206 (4.96)	160 (4.83)	112 (4.71)	22 (1.48)
Middle school	960 (23.10)	778 (22.94)	496 (20.84)	251 (16.90)
High school	897 (21.58)	756 (22.29)	473 (19.87)	330 (22.22)
Junior college	907 (21.82)	747 (22.00)	546 (22.94)	370 (24.92)
Undergraduate	1,025 (24.66)	831 (24.40)	650 (27.31)	435 (29.29)
Masters or above	161 (3.88)	120 (3.54)	103 (4.33)	77 (5.19)
Satisfaction with distance learning				
Yes	3,632 (87.39)	2,971 (87.59)	2,108 (88.57)	1,330 (89.56)
No	524 (12.61)	421 (12.41)	272 (11.43)	155 (10.44)
No procrastination	2,516 (60.50)	1,814 (53.06)	1,146 (48.15)	780 (52.53)
Moderate	1,280 (30.79)	1,155 (34.04)	990 (41.60)	550 (37.04)
Severe	362 (8.71)	454 (12.90)	244 (10.25)	155 (10.43)

### Overall changes in procrastination during the pandemic

**Figure 1:**
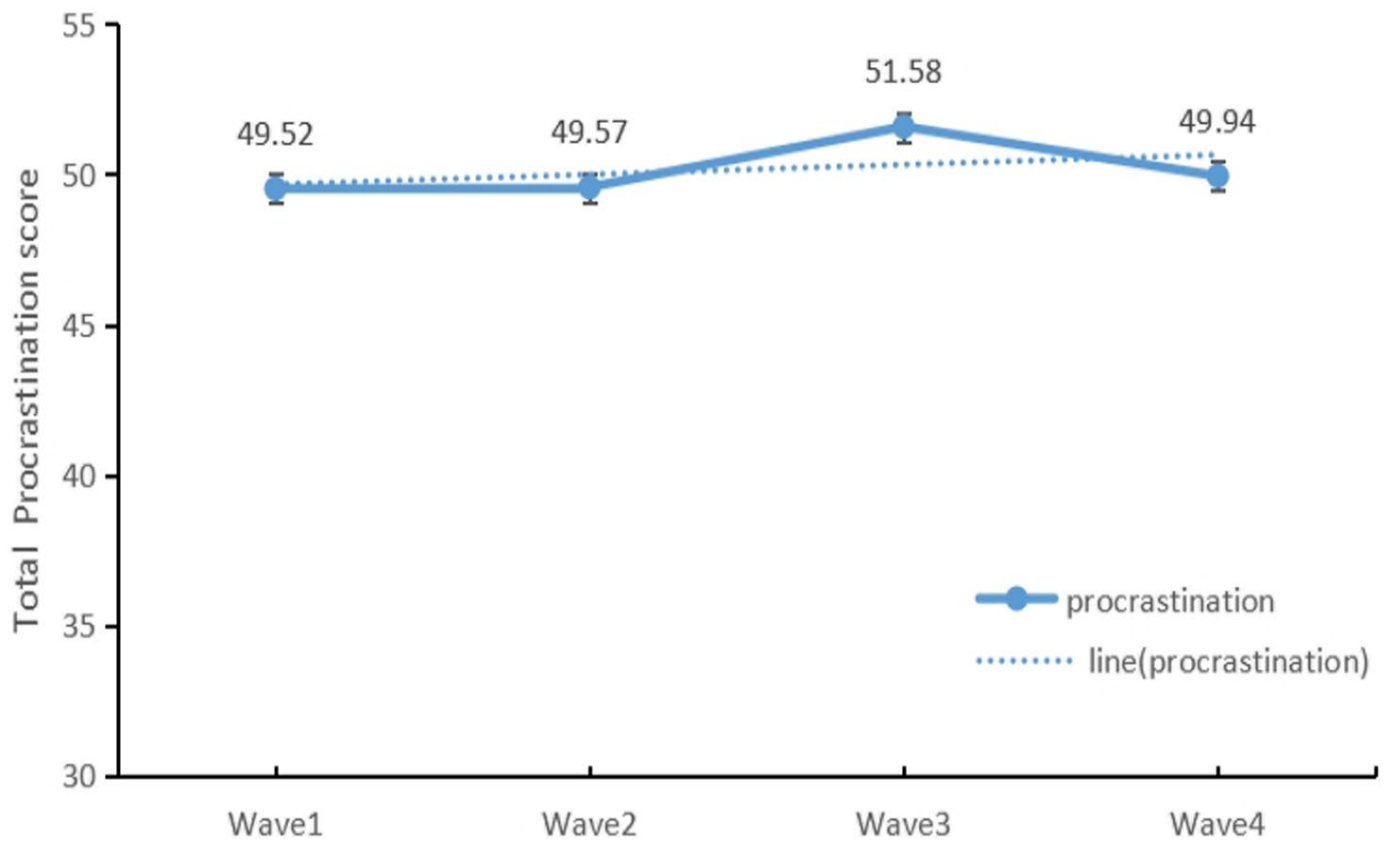
Change in estimated means for the GP Scale between Wave 1 and Wave 4.

The LGC analyses explored the overall changes in adolescents’ procrastination during the pandemic (see Figure 1). During the four waves of the COVID-19 web survey, the mean GPS-20 score overall rose, reaching a peak of 51.58 in Wave 3, then slightly declined, but was still above baseline levels. The model fitting was considered to be CFI = 0.916, TLI = 0.900, RMSEA = 0.080.

### Predictors of baseline and change over time in adolescents for procrastination

The results found that a higher grade, males, having siblings, dissatisfaction with distance learning, lower goal focus and emotional control, higher negative coping, lower income households, lower parental emotional warmth and higher parental over-protection, lower frequency exercise weekly, more daily leisure screen-time, and little online communication with peers were reported to lead to higher baseline levels of adolescents’ procrastination. Many of the variables that were associated with elevated scores at baseline were associated with a slower rate of increase in symptoms over the follow-up period. This was the case for adolescents who showed dissatisfaction with distance learning, lower levels of goal focus and emotional control, higher levels of negative coping, and daily leisure screen-time of more than 5 h. Conversely, the higher the level of parental over-protection, the faster the rate of procrastination development (see Table 2).

**Table 2:** Predictors of baseline and change over time in adolescents for procrastination.

	*B*	SE	95%CI	*p* value
Predictors of the intercept				
Age	−0.03	0.03	−0.08 to 0.02	0.276
Grade	0.06	0.03	0.01 to 0.11	0.024
Female (vs. male)	−0.08	0.02	−0.11 to 0.05	<0.001
Siblings (vs. no siblings)	0.04	0.02	0.01 to 0.08	0.009
Household economy good	−0.04	0.02	−0.07 to 0.003	0.035
Maternal high education	0.04	0.02	−0.001 to 0.07	0.056
Dissatisfaction with distance learning (vs. satisfaction)	0.14	0.02	0.10 to 0.17	<0.001
Goal focused	−0.34	0.02	−0.38 to 0.30	<0.001
Emotional control	−0.26	0.02	−0.30 to 0.23	<0.001
Passive coping	0.12	0.02	0.09 to 0.16	<0.001
Emotional warmth	−0.12	0.02	−0.16 to 0.07	<0.001
Over-protection	0.05	0.02	0.02 to 0.09	0.005
Screen: 3–5 h (vs. <3 h)	0.09	0.02	0.06 to 0.13	<0.001
Screen: ≥5 h (vs. <3 h)	0.14	0.02	0.11 to 0.18	<0.001
Exercise: barely (vs. >3 times)	0.14	0.02	0.10 to 0.18	<0.001
Exercise: <3 times (vs. >3 times)	0.06	0.02	0.02 to 0.09	0.002
Talk: barely (vs. 0–30 min.)	0.04	0.02	0.01 to 0.08	0.016
Talk: ≥30 min. (vs. 0–30 min.)	0.01	0.02	−0.03 to 0.04	0.623
Predictors of the slope				
Age	0.01	0.04	−0.08 to 0.09	0.895
Grade	0.01	0.05	−0.09 to 0.10	0.892
Female (vs. male)	0.001	0.03	−0.05 to 0.05	0.954
Siblings (vs. no siblings)	−0.04	0.03	−0.09 to 0.01	0.112
Household economy good	−0.01	0.03	−0.06 to 0.04	0.706
Maternal high education	−0.001	0.03	−0.06 to 0.06	0.977
Dissatisfaction with distance learning (vs. satisfaction)	−0.12	0.03	−0.17 to 0.06	<0.001
Goal focused	0.15	0.03	0.09 to 0.22	<0.001
Emotional control	0.08	0.03	0.02 to 0.14	0.008
Passive coping	-0.06	0.03	−0.12 to 0.01	0.030
Emotional warmth	0.03	0.03	−0.03 to 0.10	0.327
Over-protection	0.08	0.03	0.02 to 0.13	0.007
Screen: 3–5 h (vs. <3 h)	−0.02	0.03	−0.07 to 0.03	0.419
Screen: ≥5 h (vs. <3 h)	−0.07	0.03	−0.13 to 0.02	0.009
Exercise: barely (vs. >3 times)	0.02	0.03	−0.04 to 0.07	0.563
Exercise: <3 times (vs. >3 times)	0.02	0.03	−0.04 to 0.07	0.561
Talk: barely (vs. 0–30 min.)	−0.02	0.03	−0.07 to 0.04	0.515
Talk: ≥30 min. (vs. 0–30 min.)	0.01	0.03	−0.05 to 0.06	0.745

### Group-based trajectories of procrastination

**Figure 2:**
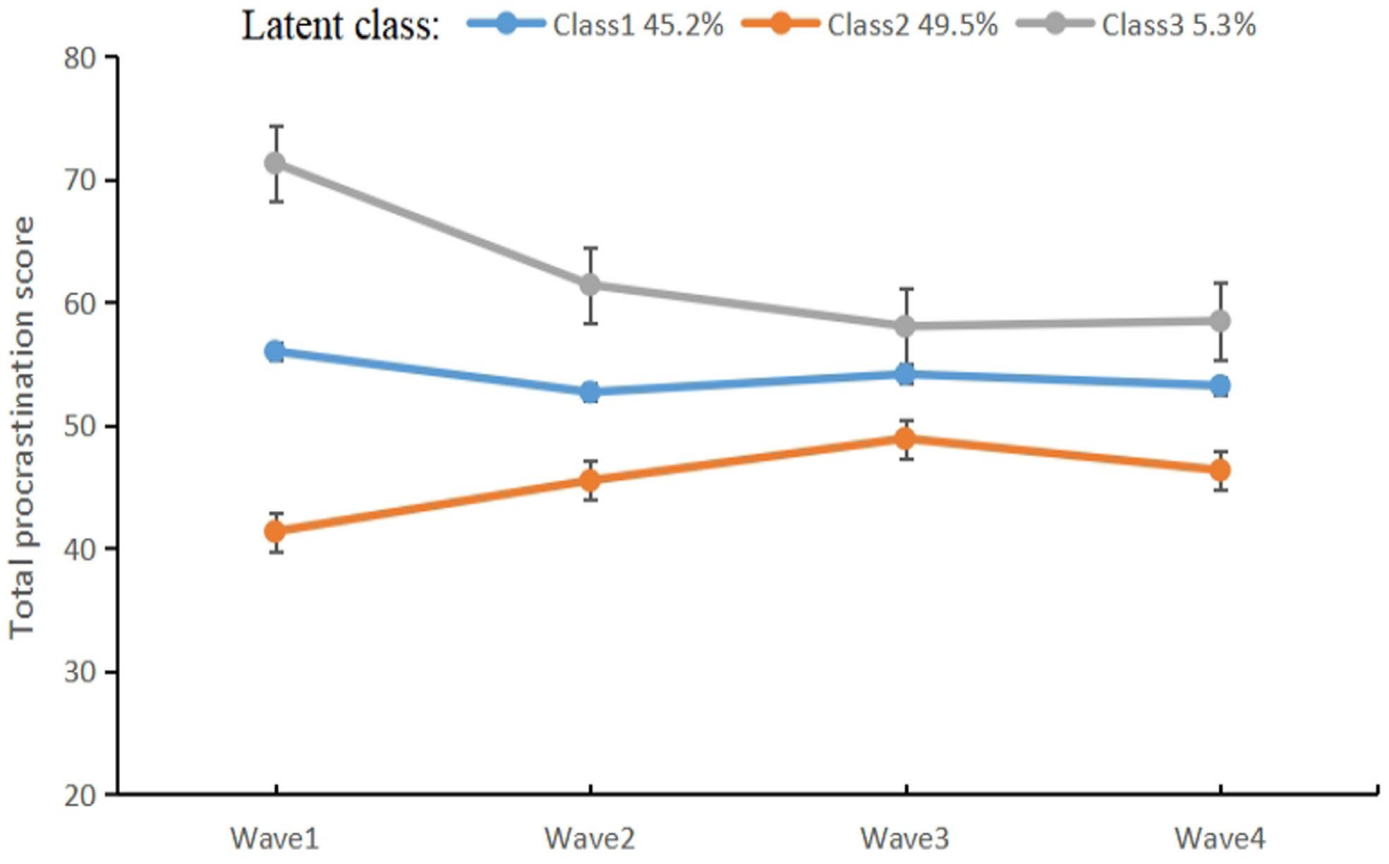
Group-based trajectories of procrastination.

After fitting models with one to six latent classes, the three-class model was considered the best fit (see Table 3). Even though models with a greater number of latent classes were associated with lower Bayesian information criterion values, the drop in Bayesian information criterion plateaued after three classes. Additionally, models with a greater number of classes were associated with considerably poorer entropy (a measure of information) and contained low-prevalence subclasses of the smaller model. Therefore, we opted for the more parsimonious three-class model of procrastination in Figure 2: Class 1, indicating moderate-stable procrastination [including 1,879 participants (45.2%)], which had moderate procrastination and remained stable over time; Class 2, low-increasing procrastination [including 2,057 participants (49.5%)], which had low procrastination but increased over time; and Class 3, high-decreasing procrastination [including 220 participants (5.3%)], which started with high procrastination but decreased over time. More information on the trajectory groups of procrastination is provided in Supplementary Tables S4, S5 of Supplemental materials.

**Table 3:** Fit statistics for 1- to 6-class models.

Model	AIC	BIC	aBIC	Entropy	LMR LR *p*-value	*N* classes >5%
1-class	85821.37	85891.03	85856.08	–	–	Yes
2-class	85722.97	85811.63	85767.14	0.56	0.047	Yes
3-class	85408.64	85516.29	85462.27	0.80	<0.0001	Yes
4-class	85353.57	85480.22	85416.67	0.82	0.081	No
5-class	85307.33	85452.97	85379.89	0.82	<0.0001	No
6-class	85246.31	85410.945	85328.328	0.78	0.371	No

### Adolescent’s risk factors for moderate-stable and high-decreasing procrastination

**Figure 3:**
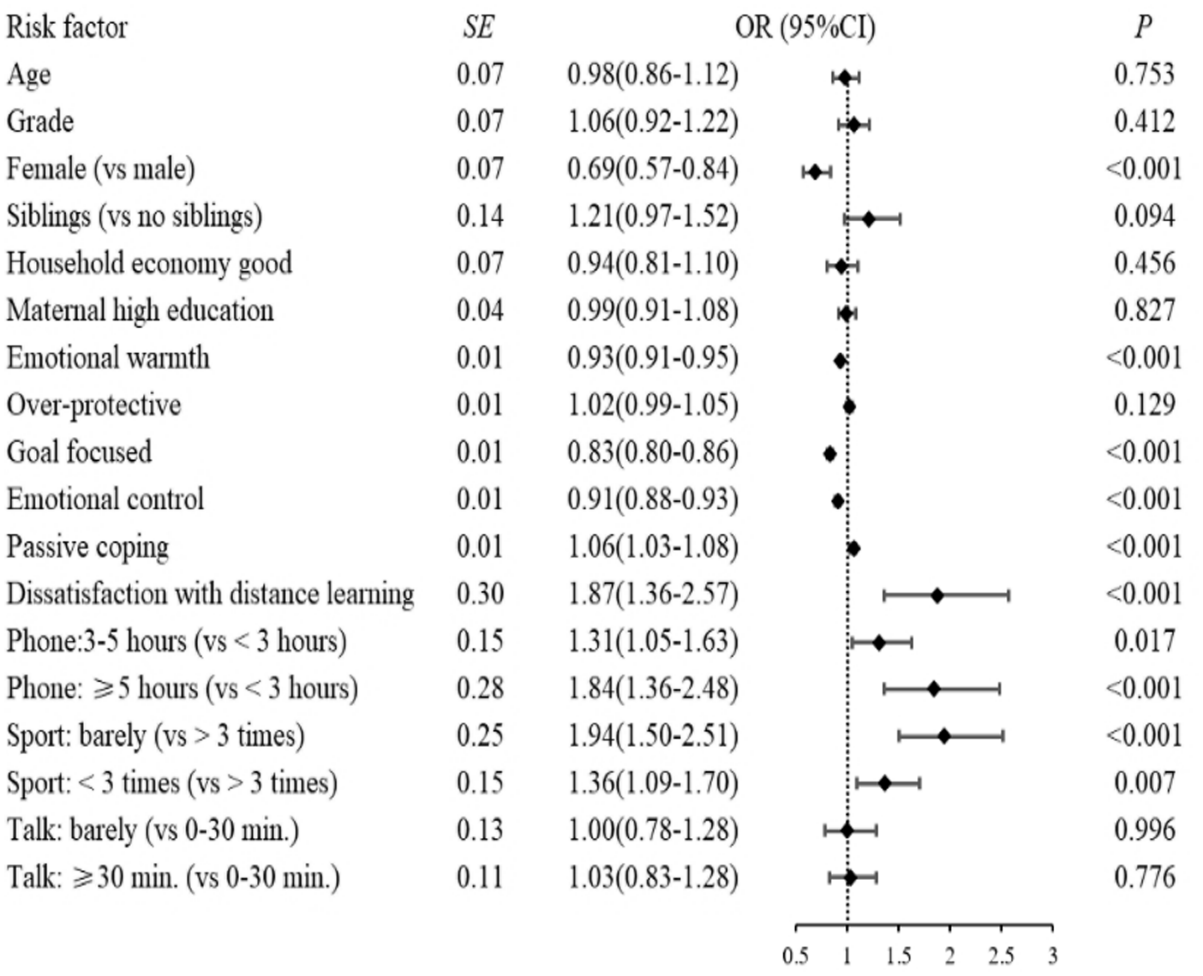
Associations of risk factors with moderate-stable procrastination.

The associations of the risk factors with the levels of severity of procrastination are presented in Figure 3. It was worth noting that more leisure screen-time daily, lower frequency of exercise weekly, and dissatisfaction with distance learning were the top three risk factors for moderate-stable and high-decreasing procrastination compared to low-increasing procrastination. Specifically, compared with a daily leisure screen-time of less than 3 h, adolescents who had more than 5 h were more likely to have moderate-stable (OR, 1.84; 95% CI, 1.36–2.48) or high-decreasing procrastination (OR, 6.39; 95% CI, 3.63–11.26). Adolescents showing dissatisfaction with distance learning were more likely to experience moderate-stable (OR, 1.87; 95% CI, 1.36–2.57) or high-decreasing procrastination (OR, 5.02; 95% CI, 3.07–8.22). Adolescents who had little exercise were more likely to be moderate-stable (OR, 1.94; 95% CI, 1.50–2.51) and high-decreasing procrastination (OR, 4.45; 95% CI, 2.44–8.12) than those who exercised more than three times a week.

**Figure 4:**
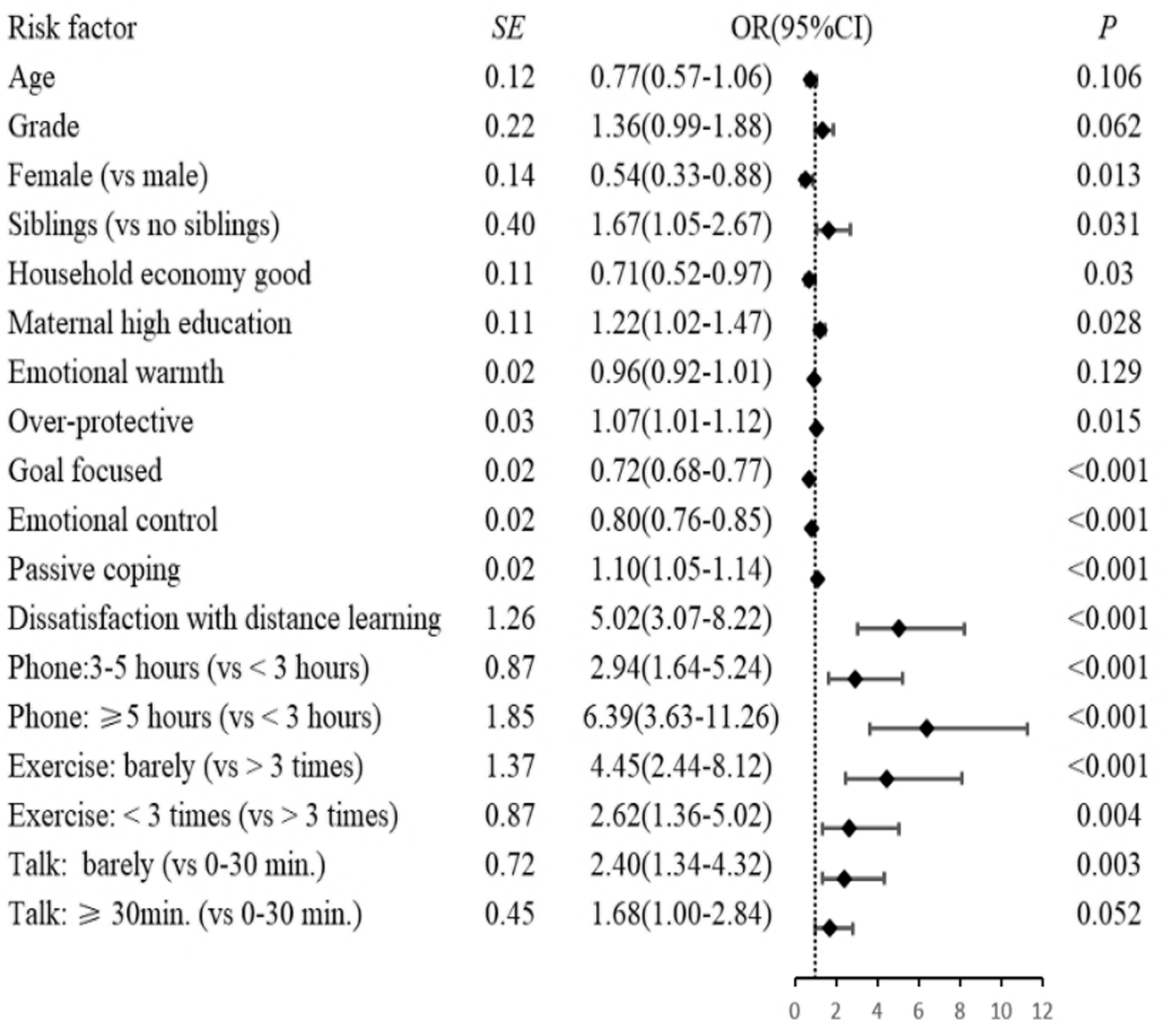
Associations of risk factors with the high-decreasing procrastination.

Compared to the low-increasing procrastination level, adolescents having siblings, adolescents with higher levels of parent over-protection, adolescents from the poorer income households, adolescents whose mothers had a higher education, and adolescents who had little daily communication were associated with a greater risk of high-decreasing procrastination but not moderate-stable procrastination. Also, the parents of adolescents with lower levels of parental warmth were more likely to suffer from moderate-stable procrastination when compared to low-increasing procrastination but not high-decreasing procrastination (see Figure 4).

## Discussion

Our results revealed that the proportion and overall trends in adolescent procrastination increased with the pandemic. A slow increase in procrastination was observed from Wave 1 to Wave 2 before the peaking in Wave 3, nearly 2 years into the pandemic, and highlights the concern that the prolonged and repeated measures may have had a negative impact on adolescents in terms of increased risk for procrastination. Further analysis revealed that around half of the participants had low-increasing procrastination and 45.2% were moderate-stable procrastination; a minority of participants (5.3%) reported a very different experience, with high-decreasing procrastination. So far, changes in the symptoms of procrastination have been inconclusive, and few studies have looked at changes in procrastination over a longer period of time (Chen et al., 2020; Diotaiuti et al., 2021a). The findings prompt schools and parents to identify and intervene in a timely manner for adolescents’ procrastination.

This study found that higher grades were predictors of procrastination, which is similar to previous studies (Steel, 2007). Due to the fact that as grade levels increase, more classroom tasks become more difficult, which leads to more procrastination behaviors. On the other hand, the higher the grade level, the more adolescents go through a period of self-awareness transition and experience more negative emotions and inadequate coping skills, causing anxiety, depression, and aversion to tasks, which in turn cause increased procrastination behaviors (Rebetez et al., 2018).

The present study found that the level of procrastination was significantly higher in males than in females, which may be due to the fact that females tend to develop earlier in terms of intelligence and cognition and have higher self-control and resistance to external temptations (Zhou, 2019). In addition to this, with different gender role expectations, females tend to be more able to manage themselves more strictly and with lower procrastination than males (de la Fuente et al., 2021).

Adolescents with siblings have much more possibility to procrastinate. Due to the special national conditions and pandemic background in China, during the lockdown period of the pandemic, both parents and children had to stay at home to work and study. Although the traditional view is that only children are selfish, maladjusted, and spoiled in China (Shi et al., 2021), during home quarantine, parents of only children would focus their attention on their only children and effectively supervise their study and life, instead of dividing the energy between other children.

Moreover, adolescents who thought their families were financially disadvantaged were more likely to develop procrastination. The pandemic has caused schools, factories, and enterprises to suspend or close, which has had a severe negative impact on societies and economies all over the world (Cucinotta and Vanelli, 2020). It has a negative impact on the economic income of some families, leading to a decline in the quality of life, and also has a negative impact on adolescents which triggers negative emotions and leads to procrastination.

Individuals with favorable mental resilience are less likely to be severely procrastinate. Specifically, procrastination reflects giving priority to short-term mental repair over achieving long-term goals, avoiding the changes that brought by stressful events as a form of self-regulation failure (Pelikan et al., 2021). Goal focused and emotional control allow people to thrive in adversity and have the ability to take action (Diotaiuti et al., 2021b). Good psychological resilience has a preventive effect on adolescent procrastination during the COVID-19. Schools and parents should make a conscious effort to improve their goal focused and emotional control.

This study revealed that a negative coping style was significantly related to adolescent procrastination, which was consistent with the results of previous studies (Rebetez et al., 2018). The sudden mass outbreak of COVID-19 has brought inevitable stress to adolescents. To a certain extent, a teenager’s coping style determines whether the individual can avoid the huge negative impact brought by the sudden outbreak. When faced with a variety of “temptations,” such as irregular study and rest time, playing truant, and eating junk food, adolescents can easily be drawn in and contribute to procrastination.

Parenting style influenced procrastination in this study. Specifically, emotional warmth and parental over-protection predicted the trend of procrastination. This is the same as the result of a few related studies, which also revealed that parenting styles can affect individual procrastination behavior, although different measuring tools were used (Khalid et al., 2019). Adolescents who experienced a parenting style of emotional warmth could better recover emotionally when faced with inevitable trauma, mitigating the negative effects of the pandemic of COVID-19 as well as the related changes in learning and lifestyle, thus avoiding procrastination. Conversely, the parenting style of over-protection encourages truancy, sleep deprivation, irregular eating, and other procrastinate behaviors during home isolation (Pychyl et al., 2002).

Adolescents who used phones more and exercised less were more likely to procrastinate (Narisada and Suzuki, 2019; Shi et al., 2021). Excessive use of mobile phones might also increase the risk of phone addiction among adolescents, as well as affect their physical and mental health, academic performance, and well-being (Diotaiuti et al., 2022). Research has shown that frequent mobile phone use consumed learning time and postponed learning tasks (Zhen et al., 2020). In particular, the time of adolescent mobile phone use during the COVID-19 pandemic was different from that in normal school hours, and adolescents were more prone to be procrastinate. Meanwhile, some research has also revealed that insufficient physical activity was significantly correlated with irrational procrastination among students (Zhong, 2013). The idea that exercise improves mood has been supported by a number of previous studies (Ferrara et al., 2022; Galli et al., 2022). Exercise can effectively protect individuals from the negative mood and emotions brought about by COVID-19 (D'Oliveira et al., 2022).

Dissatisfaction with distance learning will lead to adolescent procrastination. The outbreak of COVID-19 has prompted the education departments of all countries to advocate online and distance learning (Melgaard et al., 2022). However, as a new way of teaching, distance learning is less supervisory than face-to-face teaching, and the effectiveness of online learning is still questioned (Huang et al., 2020). Furthermore, the insufficient adjustment ability and poor adaptability of adolescents may be an influencing factor for procrastination (Mohammadi Bytamar et al., 2020).

In addition, it is worth indicating that adolescents with mothers who had a higher level of education were more likely to develop severe procrastination. Previous studies have shown that highly educated women have generally moved away from family roles and living arrangements to pursue their career as well as performing important social roles (Cooney and Uhlenberg, 1991). In China, the government has asked all public officials to take an active part in anti-pandemic work, such as screening close contacts, isolating patients, and conducting nucleic acid testing. This means highly educated women might be busy in public service jobs during this period, rather than teaching and caring for their children.

### Limitations of the study

Some limitations need to be considered in this study. First, we did not obtain pre-pandemic data, which makes it difficult to know the extent to which our baseline measures had already been affected by experiences of COVID-19 up to that point. Second, there is a myriad of factors that could possibly influence the wellbeing of adolescents during a pandemic, and we have only captured some of these in the present study. Third, the virtual nature of the COVID-19 survey may have weakened comparisons with data from in-person surveys, as this modality could have hindered candid reporting. Finally, the samples are not expected to be nationally representative, and the results should be interpreted with this in mind.

## Conclusion

The prospective design that allows us to confirm the development trends of adolescents’ procrastination during the COVID-19 pandemic (June 2020 to October 2021) is a clear strength of this study. To the best of our knowledge, this is the first prospective study of procrastination in adolescents during COVID-19.

Overall, our study demonstrates an increase in the proportion and trends of procrastination among adolescents affected by the pandemic, thus suggesting that the prolonged situation of the ongoing pandemic and the related restrictions have taken a toll on the adolescent population. Around half of the participants had low-increasing procrastination and 45.2% were moderate-stable procrastination throughout the study period; a minority of participants (5.3%) reported a very different experience, with high-decreasing procrastination. Efforts should be made to reach out to adolescents with interventions, especially those at high risk, calling on adolescents to do physical exercise more than three times a week and use electronic screens for leisure less than 3 hours daily. Parents should give their children proper attention and not spoil or over-protect them. Schools and education authorities should optimize online learning systems and psychological counseling to improve students’ satisfaction with distance learning.

## Data availability statement

The original contributions presented in the study are included in the article/Supplementary material, further inquiries can be directed to the corresponding authors.

## Ethics statement

The studies involving human participants were reviewed and approved by the Ethics Committee of Harbin Medical University. Written informed consent to participate in this study was provided by the participants’ legal guardian/next of kin.

## Author contributions

YoW ran the statistical models and wrote the manuscript. TB revised the manuscript. YX collected and cleared the data. PW and JXZ conducted the preliminary data collation. KQ and YaW performed the survey administration. LC, JY, XQ, and XY contacted the schools and organizations involved in the study. YY and ZQ oversaw the project, collected data, and contributed financial backing. All authors contributed to the article and approved the submitted version.

## Funding

This research was supported by the National Natural Science Foundation of China (81773536) to YY and the Natural Science Foundation of Heilongjiang Province, China (LH2021H005) to ZQ.

## Conflict of interest

The authors declare that the research was conducted in the absence of any commercial or financial relationships that could be construed as a potential conflict of interest.

## Publisher’s note

All claims expressed in this article are solely those of the authors and do not necessarily represent those of their affiliated organizations, or those of the publisher, the editors and the reviewers. Any product that may be evaluated in this article, or claim that may be made by its manufacturer, is not guaranteed or endorsed by the publisher.

## Supplementary material

The Supplementary material for this article can be found online at: https://www.frontiersin.org/articles/10.3389/fpsyg.2023.1168463/full#supplementary-material

## Footnotes

^1^ www.wjx.cn
