# Supplementary material for: Trajectories and influencing factors in adolescent procrastination behavior throughout the COVID-19 pandemic: a four-wave prospective longitudinal study
Source: Front Psychol. 2023 Jun 22;14:1168463. doi: 10.3389/fpsyg.2023.1168463 (PMC10324606; doi:10.3389/fpsyg.2023.1168463)
Supplement: Supplementary file 1 [file Table_1.DOCX]

Supplemental materials

Table S1. Patterns of non-response for 4156 respondents to the COVID-19 web-survey

| Variable | N | % |
| --- | --- | --- |
| **Missing at each wave** |  |  |
| Wave2 | 764 | 18.38% |
| Wave3 | 1776 | 42.73% |
| Wave4 | 2671 | 64.27% |
| **Missing for number of waves** |  |  |
| No waves | 925 | 22.26% |
| 1 wave only | 1251 | 30.10% |
| 2 waves | 1980 | 47.64% |
| **Patterns of non-response** |  |  |
| All complete | 925 | 22.26% |
| Wave 4 only | 785 | 18.89% |
| Wave 3 only | 149 | 3.59% |
| Wave 3 & 4 | 1533 | 36.89% |
| Wave 2 only | 317 | 7.63% |
| Wave 2 & 3 | 94 | 2.26% |
| Wave 2 & 4 | 353 | 8.49% |

Table S2. Characteristics of participants who were excluded from the analysis due to missing data.

| Excluded participants (N=1395) | N % | |
| --- | --- | --- |
| **Gender** |  |  |
| Male | 673 | 48.24 |
| Female | 722 | 51.76 |
| **Age, year** |  |  |
| 11-12 | 338 | 24.23 |
| 13-14 | 930 | 66.67 |
| 15-16 | 121 | 8.67 |
| 17-18 | 6 | 0.43 |
| **Grades** |  |  |
| Junior one | 546 | 39.14 |
| Junior two | 591 | 42.37 |
| Junior three | 182 | 13.05 |
| Junior four | 44 | 3.15 |
| senior one | 31 | 2.22 |
| senior two | 1 | 0.07 |
| senior three | 546 | 39.14 |
| **Presence of siblings** |  |  |
| No siblings | 1093 | 78.35 |
| siblings | 302 | 21.65 |
| **Household economy** |  |  |
| Very poor | 13 | 0.93 |
| Poor | 9 | 0.65 |
| General | 783 | 56.13 |
| Good | 433 | 31.04 |
| Very good | 157 | 11.25 |
| **Maternal education** |  |  |
| Primary and below | 34 | 2.44 |
| Middle school | 230 | 16.49 |
| High school | 308 | 22.08 |
| junior college | 353 | 25.30 |
| undergraduate | 408 | 29.25 |
| Master or above | 62 | 4.44 |
| **Satisfaction of distance learning** |  |  |
| Yes | 1345 | 96.42 |
| No | 50 | 3.58 |

Table S3. Observed Procrastination Scores at Each Wave by Latent Trajectory Group Membership

| Wave | No procrastination | | Moderate | | Severe | |
| --- | --- | --- | --- | --- | --- | --- |
|  | *Mean* | *SD* | *Mean* | *SD* | *Mean* | *SD* |
| Wave1 | 40.95 | 4.29 | 56.31 | 4.62 | 72.78 | 5.01 |
| Wave2 | 45.30 | 11.51 | 52.79 | 11.20 | 62.51 | 12.57 |
| Wave3 | 48.75 | 10.19 | 54.22 | 10.27 | 58.44 | 11.02 |
| Wave4 | 45.99 | 11.48 | 53.48 | 11.27 | 59.10 | 13.16 |

Note. The No procrastination trajectory (Class 2) includes participants with minimal procrastination at all waves [2057 individuals (49.5%)]. The Moderate procrastination trajectory (Class 1) represents participants with moderate procrastination throughout the study [1879 individuals (45.2%)]; The procrastination trajectory (Class 3) represents participants with persistently severe levels of procrastination [220 individuals (5.3%)].

Table S4. Sample breakdown and intercept and slope coefficients by trajectory group

| Trajectory | *N* (%) | Intercept | Slope | *P* |
| --- | --- | --- | --- | --- |
| Severe | 220(5.3) | 71.25 | -9.52 | <0.001 |
| Moderate | 1879(45.2) | 55.92 | -1.92 | <0.001 |
| No procrastination ^a^ | 2057(49.5) | 41.29 | 4.89 | <0.001 |

^a^Reference groups.
